# *In-situ* monitoring of polymer mechanochemistry: what can be learned from small molecule systems

**DOI:** 10.3389/fchem.2024.1490847

**Published:** 2024-10-16

**Authors:** Niamh Willis-Fox

**Affiliations:** Department of Materials, The University of Manchester, Manchester, United Kingdom

**Keywords:** mechanochemistry, *in-situ* monitoring, scale up, ball milling, polymer mechanochemistry

## Abstract

Using mechanical energy to drive chemical transformations is an exciting prospect to improve the sustainability of chemical reactions and to produce products not achievable by more traditional methods. *In-situ* monitoring of reaction pathways and chemical transformations is vital to deliver the reproducible results required for scale up to realize the potential of mechanochemistry beyond the chemistry lab. This mini review will discuss the recent advances in *in-situ* monitoring of ball milling and polymer mechanochemistry, highlighting the potential for shared knowledge for scale up.

## 1 Introduction

The use of mechanical energy to drive chemical transformation is a burgeoning field which has given rise to chemical products unachievable via traditional methods, while at the same time using processes with dramatically improved sustainability, for example, using solvent-free conditions (Friščić et al., 2020). This has led IUPAC to name mechanochemistry among the top ten emerging technologies (Gomollón-Bel, 2019). The usefulness of mechanochemistry has been proven in the chemistry lab, both in synthesizing smaller molecules during ball milling (BM) and in driving productive transformations in polymer materials. Despite the immense promise of mechanochemistry, a major current challenge with its adoption is understanding how these chemical transformations can be scaled up beyond the lab bench towards industrial processes (Reynes et al., 2023; Willis-Fox et al., 2023). Scale up is not a linear process which simply involves using larger ball milling equipment. As highlighted by Reynes et al. (2023), a number of vastly different considerations must be taken into account such as heat evolution, competition with established methodologies and standardized testing. Similar challenges are also a concern for the scale up of polymer mechanochemistry (PM) in which the understanding gained on a single polymer chain level does not linearly scale into bulk material due to inhomogeneity in polymer networks (Willis-Fox et al., 2023). Overcoming these barriers and optimizing mechanochemical transformations for industrial scales will require knowledge of the specific reaction processes and how these are altered during realistic conditions for scale up.

As highlighted by O’Neill and Boulatov (2021), the use of mechanical energy to drive chemical change encompasses diverse phenomena ranging from motor protein function, organic synthesis in a ball mill, and polymer fragmentation in ultrasonication (US) and in mastication (O’Neill and Boulatov, 2021). The sub-fields studying each of these manifestations of mechanochemistry have developed individual practices and techniques such as BM and ultrasonication (US) traditionally used for mechanochemical synthesis and investigation of PM, respectively. However, the distinction between various expressions of mechanochemistry is blurring; for example, depolymerization of commodity polymers and mechanoredox polymerization have recently been displayed in both BM (Jung et al., 2024; Skala et al., 2024) and US. (Young et al., 2024; Zhou et al., 2023; Zeitler et al., 2024) Despite this blurring, the field remains vast, and thus this mini review will focus on two of the most commonly examined areas: mechanochemical synthesis via BM and chemical transformations brought on via PM, with the acknowledgement that more nuanced discussions of each topic are available through reviews highlighted below and elsewhere.

A particular difficulty for scale up is that mechanochemical reactions usually occur within a sealed vessel, meaning the chemical transformation must be inferred from post-processing analysis. The field of BM addresses this through the development of *in-situ* techniques to probe the transformations as they occur during the process of milling, most frequently using shaker mills (Michalchuk and Emmerling, 2022). In polymer mechanochemistry (PM) there are a wider range of techniques used to input mechanical force such as single molecule force spectroscopy (SMFS), ultrasonication (US) and extension or compression of bulk materials. This makes it harder to develop standardized techniques to monitor chemical transformations in PM.

Thus, although the approaches to induce mechanochemical transformations differ between BM and PM (Aydonat et al., 2024), the common goal of using mechanical energy to drive productive chemical transformations suggests the possibility of shared lessons on the scale up journey. This mini review aims to highlight the concepts for *in-situ* monitoring for both small molecule and polymer mechanochemistry, discuss recent advances in each, and, finally, explore the potential for shared lessons.

## 2 *In-situ* monitoring for scale up of mechanochemical synthesis

### 2.1 Lab-scale mechanochemical transformations

There are many methods to induce mechanochemical transformations on a lab scale such as ball milling, resonant acoustic mixing (RAM) and twin-screw extrusion (TSE). Although RAM and TSE are rapidly gaining popularity, BM remains the most widespread technique for mechanochemical synthesis. During BM, mechanical energy is introduced through collisions between particles and the milling media. Various types and scales of mill are commonly used to perform mechanochemical reactions, such as vibratory mills, planetary mills and SPEX mills (Fantozzi et al., 2023). Reynes et al. (2023) have discussed transformations demonstrated in each of these milling media and highlighted the positive implications for scale-up of sustainable mechanochemical synthesis. BM offers the advantage of an enclosed reaction environment with well-defined parameters (Friščić et al., 2020). This importantly gives rise to the reproducibility required for scale up. However, the enclosed nature of the reaction vessels made from stainless steel or other opaque materials restricts the potential to probe the mechanism of the mechanochemical reaction as it progresses (Michalchuk and Emmerling, 2022; Boldyreva, 2022). Thus, to optimize these reactions beyond trial and error, it is important to develop methods to probe the reaction *in-situ*.

### 2.2 Time-resolved *in-situ* (TRIS) monitoring

**FIGURE 1:**
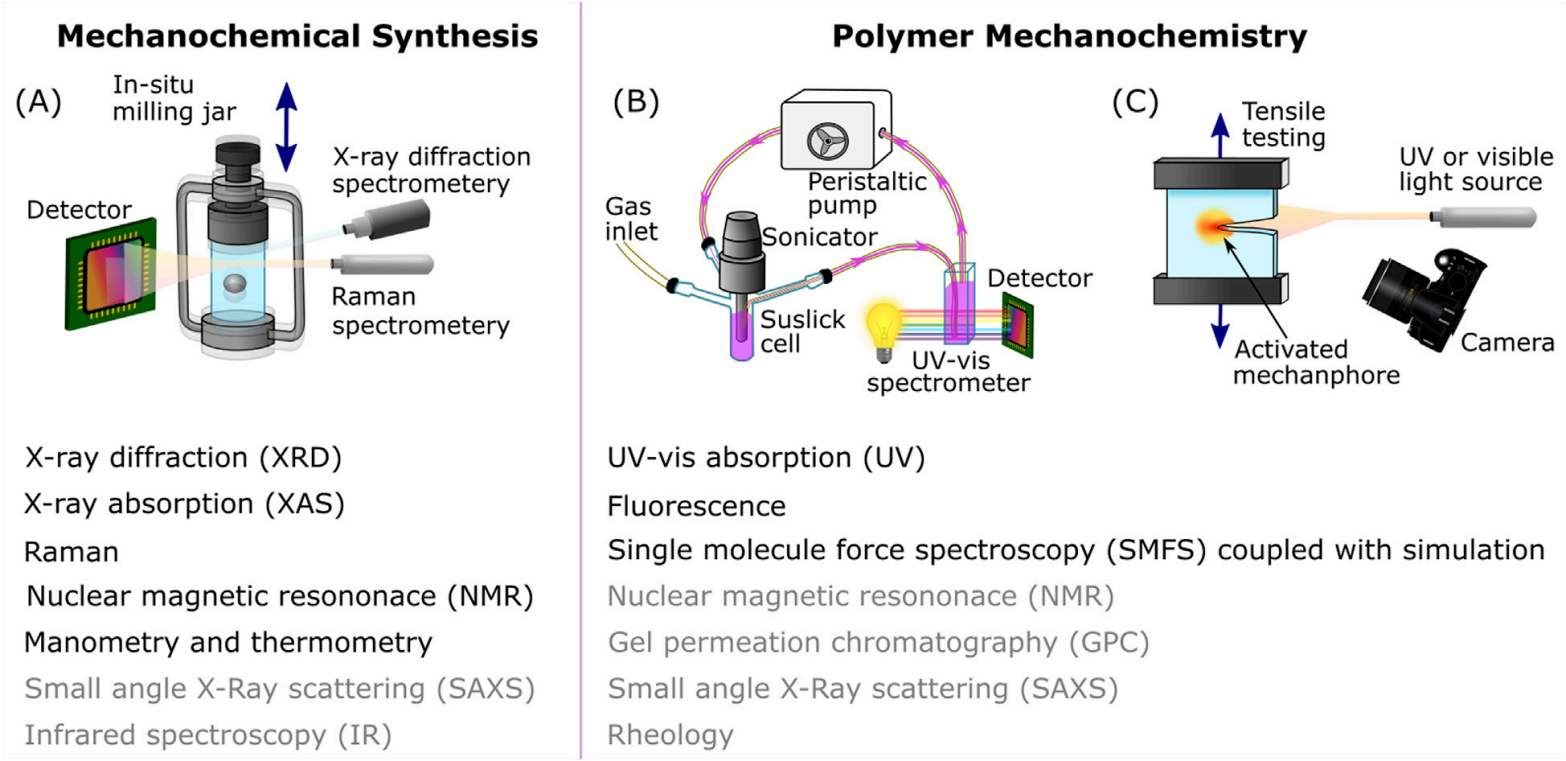
Schematic representation of time-resolved *in-situ* measurements for mechanochemical synthesis and polymer mechanochemistry; **(A)** TRIS during ball milling, **(B)** *In-situ* UV-vis absorption of polymer mechanochemistry in solution and **(C)** *in-situ* imaging of mechanophore activation during tensile testing. Including established (in black) and potential (in grey) TRIS characterization methods for mechanochemical synthesis and polymer mechanochemistry. Schematic **(A)** is adapted from ref (O’Neill and Boulatov, 2021). Schematic **(B)** is adapted from ref (Muramatsu et al., 2023).

The vast majority of BM reactions are analyzed once the mechanical treatment is finished as removing material from the reactor can alter the course of the reaction (Michalchuk and Emmerling, 2022; Michalchuk et al., 2013). Therefore, the idea of following mechanochemical transformations through time-resolved *in situ* (TRIS) methods has gained popularity. Recently Michalchuk and Emmerling have provided an excellent review of the TRIS literature, outlining techniques to monitor BM reactions and the information that can be gained from each one (Michalchuk and Emmerling, 2022). As shown in Figure 1, these techniques include manometry, thermometry, X-ray diffraction, and vibrational, nuclear magnetic resonance and X-ray absorption spectroscopy. Recently, Aydonat and coworkers have further discussed these *in-situ* techniques in the context of polymer degradation (Aydonat et al., 2024). Due to the solid-state nature of the reactants and products during milling, X-ray diffraction (XRD) is a powerful tool for detecting reaction pathways and intermediates that are undetectable via *ex-situ* characterization methods (Scheurrell et al., 2023). Despite its expanding use, there is still much to be learned about *in-situ* XRD, in particular how to deal with the reduction in data quality for *in-situ* measurements compared to *ex-situ* experiments. The Emmerling group have done much work to improve data fitting (Macchietti et al., 2024) and quantification of the contribution of the instrument resolution function to remove the reliance on jar alignment (Mazzeo et al., 2023).

A way to overcome the loss in data quality for *in-situ* measurements is to couple multiple measurements in a single study. The Friščić group have recently demonstrated the dual use of time-resolved fluorescence emission and Raman spectroscopy to monitor cocrystallization of active pharmaceutical ingredients (Julien et al., 2023). This is an exciting development for benchtop experimental set-ups as it allows monitoring of amorphous phases, which is not possible using XRD. This study also uses periodic density functional theory (DFT) calculations to rationalize the TRIS fluorescence experiments. A range of theoretical techniques have been developed to probe molecular-level mechanisms of mechanochemical processes that cannot be obtained experimentally (Pladevall et al., 2021; Ferguson and Friščić, 2024; Lukin et al., 2022). Most recently, discrete element method (DEM) simulations have been used to predict the monomer yield following the depolymerisation of poly(ethylene terephthalate) (PET) by BM (Anglou et al., 2024). TRIS is not limited to BM alone. Resonant acoustic mixing (RAM) is a technique gaining much interest due to its ability to provide intensive mixing of powders without the need for milling media which facilitates scale up (Lennox et al., 2023). Michalchuk et al. (2018) exploited TRIS XRD during RAM to monitor the cocrystal formation of form III of carbamazepine and form I of nicotinamide. Extending the use of TRIS beyond traditional BM is an exciting prospect that has the potential to apply to scale up of other mechanochemical technologies.

### 2.3 Scale up

Taking a mechanochemical process from a lab scale through scale up to industrial settings is not a linear process (Reynes et al., 2023; Willis-Fox et al., 2023). Considering the dependence on milling media, jar design and the mill itself, scale up will involve many considerations which may be monitored by *in-situ* experiments. Recently, TRIS XRD and Raman spectroscopy of small scale milling reactions in a shaker mill showed the importance of water in the synthesis of calcium urea sulfate cocrystals (Brekalo et al., 2022). The understanding gained on a small scale allowed the authors to develop reaction conditions which lead to full conversions in a planetary mixer.

Along with reactor size, another issue with mechanochemical scale up is heat management, as many reactions require certain operating temperatures to drive the desired transformations (Reynes et al., 2023). The Michalchuk group have developed temperature-controlled BM set-ups with TRIS monitoring capabilities. They have demonstrated that the conversions between Form II and Form I of various organic cocrystals occur at lower temperatures during BM compared to their conventional thermodynamic transition point (Linberg et al., 2023; Linberg et al., 2024). The temperature control of such a system will be vital for the reliable scale up of mechanochemistry. The concomitant TRIS information gathered highlights the energy saving potential for mechanochemistry scale up, with desired crystal forms reached at markedly lower temperatures.

### 2.4 Twin screw extrusion (TSE)

**FIGURE 2:**
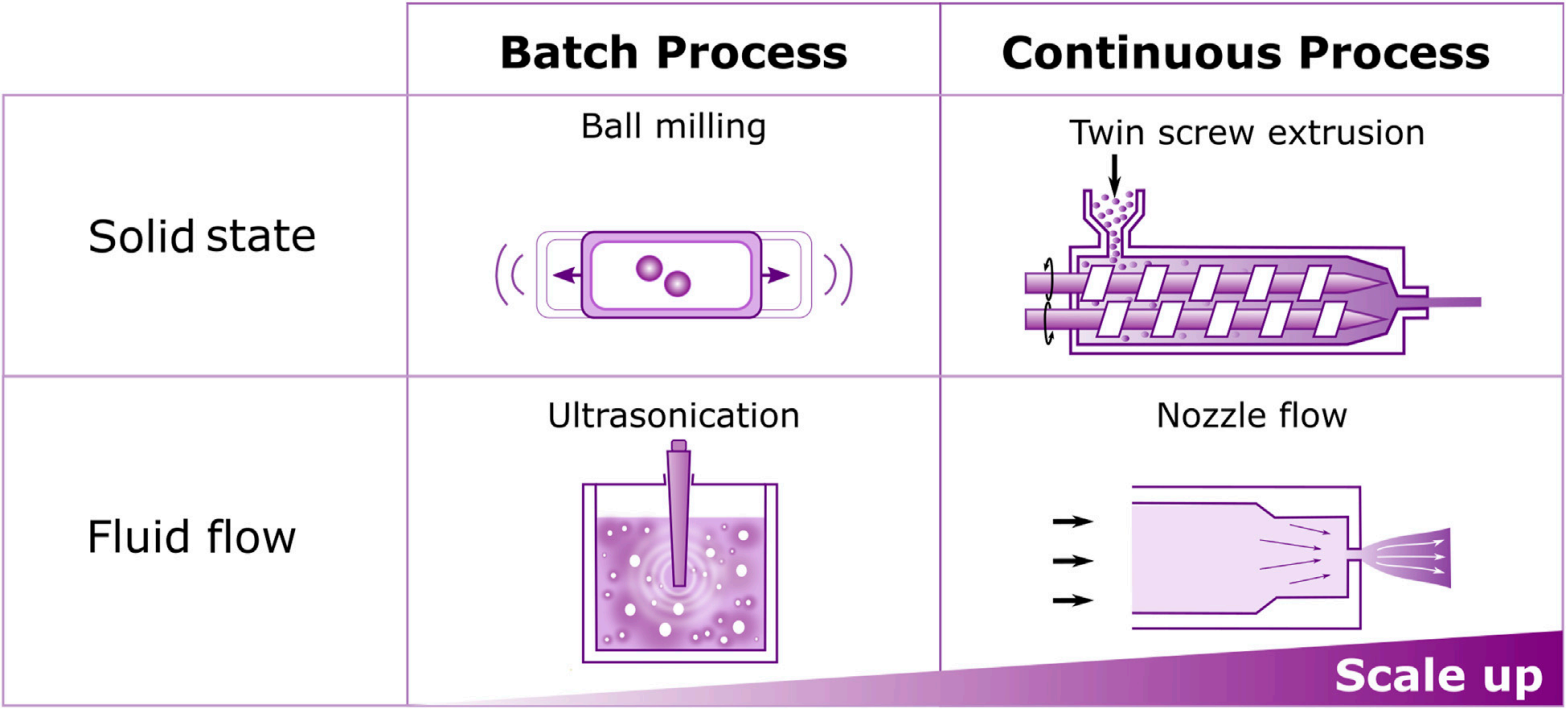
Schematic representation of batch and continuous processes for driving mechanochemistry in the solid-state and in fluid flow. The transition from batch to continuous processes will aid scale up of mechanochemistry to final application.

As noted above, TSE is rapidly gaining popularity for lab scale synthesis. Reactive TSE also offers great potential when considering mechanochemistry scale up as it moves from the “batch” nature of BM to continuous “flow,” as highlighted in Figure 2. Many advances have been made in TSE, with a wide variety of products produced at scale ranging from the Knoevenagel reaction between vanillin and barbituric acid (Crawford et al., 2017) to organic dyes such as perylene-3,4,9,10-tetracarboxylic acid diimines (PDIs) (Cao et al., 2020) or 2-D materials (Chen H. et al., 2024). As recently reviewed by Browne, the developments in small molecule synthesis by TSE have largely been driven by the pairing of Crawford and James (Bolt et al., 2022). Not only does TSE improve sustainability by reducing the solvent required, but also by a significant reduction in the energy required, global warming potential and terrestrial ecotoxicity as determined by a recent life cycle assessment (Galant et al., 2022).

As TSE-driven mechanochemical synthesis is in early development, *in-situ* measurements of reactive TSE processes have been limited to date. On the other hand, extrusion has long been used to process polymer melts and methods currently include in-line monitoring via UV-vis, near infrared (IR), light scattering, polarized optical microscopy, rheo-optics and ultrasonication (US) (Alig et al., 2010; Li et al., 1997; Montano-Herrera et al., 2014; Françla et al., 2000; Teixeira et al., 2014; Teixeira et al., 2021). However, as these techniques focused on quantifying polymer degradation, probes were introduced into the melt channel post-extrusion leaving them unable to elucidate reaction mechanisms. Recently, the Emmerling group have adapted their expertise in TRIS of BM to examine TSE. They developed a TSE extruder compatible with a non-contact Raman probe which allows for focusing of the laser close to the central conjunction of the two screws (Gugin et al., 2023). This TRIS set-up highlighted that the majority of reactions under examination had already occurred by the 5th section of the extruder. As Raman spectroscopy cannot provide information about phase transitions the same group developed a method to carry out energy-dispersive XRD monitoring during TSE, allowing them to monitor four different model reactions (Gugin et al., 2024). *In-situ* understanding on this level informs more economical extruder design, further enhancing the sustainability advantage of TSE in the scale up of mechanochemical synthesis.

## 3 *In-situ* monitoring for scale up of polymer mechanochemistry

### 3.1 Polymer mechanochemistry (PM)

Unlike the case of small molecule BM, mechanical energy has historically been seen as a destructive input for larger molecules. Harnessing mechanical energy to drive productive chemical transformations in polymers has been made possible through the introduction of the mechanophore (Ghanem et al., 2021; Li et al., 2015). Mechanophores are force-sensitive moieties that undergo controlled transformation following the application of stress. A wide range of mechanophores has been developed, capable of changes in color (Zong et al., 2024; Yamamoto et al., 2024; Hemmer et al., 2023), fluorescence (Kiebala et al., 2023; Chen M. et al., 2024), catalyst generation (Clough et al., 2015), or small molecule release (Zeng et al., 2024; Shi et al., 2020; Chen L. et al., 2024) reactions that lead to different functional outputs compared to those obtained via thermal reactions (Brown et al., 2021; Hickenboth et al., 2007). Chen et al. (2021) recently produced a comprehensive review of polymer mechanochemistry (PM) a field with applications in force sensing (Willis-Fox et al., 2023; Zong et al., 2024), drug delivery (Zeng et al., 2024; Fu and Hu, 2024; Fan et al., 2024) and polymer recycling (Young et al., 2024; Aydonat et al., 2024). Thus, just like mechanical synthesis of small molecules it is timely that the PM field considers scale up requirements (Willis-Fox et al., 2023).

In contrast to the supply of mechanical energy via collisions in small molecule mechanochemistry, in PM, mechanical energy is used to stretch polymer chains, eventually changing the properties of the embedded mechanophore (O’Neill and Boulatov, 2024). Novel mechanophore systems are commonly examined using techniques such as single molecule force spectroscopy (SMFS) or ultrasonication (US). SMFS offers control over the force exerted on a single polymer chain through rate and distance of cantilever retraction, while US enables spectroscopic characterization of the products of a mechanochemical reaction in bulk but with dramatically reduced control of the force experienced on a single molecule level (O’Neill and Boulatov, 2021).

### 3.2 Single molecule force spectroscopy (SMFS)

SMFS is widely used to understand single polymer chain behavior under load (Martínez-Tong et al., 2021; Yang et al., 2020; Bowser and Craig, 2018; O’Neill and Boulatov, 2022). The force between a probe and a surface is measured when a mechanophore-containing polymer is fixed between them. Retraction experiments can either be carried out at constant rate or at constant force to produce force-extension curves. SMFS alone does not give direct information regarding reaction pathways or transition states and SMFS information is often coupled with computation analysis using empirical models of mechanochemistry. Methods such as constrained geometries simulate external force (CoGEF) (Klein et al., 2020) and the recently reported tension-activated carbon-carbon bond (Sun et al., 2024a) are used to compute the reactivity of mechanophores. Models such as the Bell-Evans and Dudko-Hummer-Szabo models coupled to SMFS experiments have been used to understand charge-dipole repulsion of a cucurbit[7]uril-Hexanoate-isoquinoline complex mechanophore (Xia et al., 2024) and the solvent polarity effects on the mechanochemistry of spiropyran ring opening (Lin et al., 2024). SMFS and *ab initio* steered molecular dynamics have been used to understand the mechanism for SO_2_ release from a thermally stable mechanophore (Sun et al., 2024b). This thermally stable mechanophore is an important step forward for PM scale up, as incorporation into current polymer processing techniques will require mechanophores to be inert to competing inputs such as heat (Willis-Fox et al., 2023).

One of the clear strengths of TRIS for mechanochemical synthesis is the combination of complementary techniques to probe many facets of a transformation. As many mechanophores have an optical signal, this gives rise to the intriguing possibility of combining SMFS with single-molecule fluorescence spectroscopy. Such experiments have been developed for biological systems, but rather than using atomic force microscopy, these experiments use optical tweezers combined with fluorescence imaging in a technique known as “fleezers” (Bustamante et al., 2021). With the development of commercially available optical tweezing systems, their use for exploration of PM is an exciting prospect. A host of information on reaction pathways has been revealed through the use of SMFS coupled with computational methods. However, this necessarily focuses on mechanophore(s) in a single chain without information on how activation might scale in more realistic conditions.

### 3.3 Solid-state PM

In final PM applications, mechanophores will require incorporation into bulk polymer materials and networks. Optical force probes (OFPs) or mechanophores which undergo changes in optical properties have been used to understand the mechanical behavior of polymeric systems and have been well reviewed (Huang et al., 2023; He et al., 2021; Xuan et al., 2022). Many of these systems cause a change in fluorescence signal and so can be monitored by fluorescence or confocal microscopy. A notable point is the need for a transparent host matrix to enable the optical read out. This can be challenging when dealing with engineering plastics (Willis-Fox et al., 2023; Chen et al., 2021). OFP mechanophores have proved useful to monitor fracture behavior in polymer glasses (Aerts et al., 2023) and the effect of subcritical fracture on elastomer failure (Ju et al., 2023). The change in dimer/excimer structure is a very promising mechanophore strategy which has been used to characterize behavior in polyolefin elastomers (Micheletti et al., 2024) and a range of commodity host polymers (Kiebala et al., 2023). Due to the ubiquitous use of mechanical force in biological systems there is also a drive to utilize mechanophores to monitor the mechanical properties of biologically relevant systems, in particular hydrogels (Muramatsu et al., 2023; Yang et al., 2022; Merindol et al., 2019; Wei et al., 2023).

Mechanophore activation in bulk materials is highly dependent on local strain which in turn depends on factors such as sample dimensions and network structure (Chen et al., 2021). Therefore, the intensity of the mechanophore signal must be calibrated to the local stress, for example, through comparison with standards with known concentration of activated mechanophores. This challenge has been investigated in acrylate elastomers (Slootman et al., 2020; Chen et al., 2020), PDMS (Rencheck et al., 2022), poly (methyl methacrylate) (Celestine et al., 2019) and fiber-reinforced composites (Haque et al., 2023). Developing universal and reliable calibration methods is vital to use PM for *in-situ* monitoring of bulk materials.

### 3.4 Ultrasonication (US)

US is one of the most common techniques to probe novel mechanophore transformations. In brief, during US the collapse of cavitation bubbles creates elongational flow in the solution that stretches the polymer chains, activating the embedded mechanophores. Polymer chain stretching in such flows is dependent on the dynamics of the system (O’Neill and Boulatov, 2024). Thus, US is used to determine the kinetics of the mechanochemical transformation of interest. In general, reaction mixture aliquots are removed at various time points and analyzed offline. However, the Moore group have developed an experimental set-up to quantify UV-vis absorption changes in real time using a peristaltic pump to continuously transport the reaction solution through a UV-vis flow cell and return it to the reaction vessel (May et al., 2016). Similar systems have been used to examine the properties of naphthopyran mechanophores (McFadden and Robb, 2019; Osler et al., 2021; McFadden et al., 2022), the generation of a permanent merocyanine species which cannot be depleted thermally (McFadden and Robb, 2021) and the investigation of spin traps to follow mechanochemical scission of polymer chains which do not contain mechanophores (Wang et al., 2017).

Similar to BM, PM products can be transient or even revert to their original state after stress is removed; thus, the concept of radical traps is an intriguing one for examining PM reaction pathways. The Craig group used this strategy to trap a diradical that is formally a transition state in the mechanical ring opening of the gem-difluorocyclopropane mechanophore (Lenhardt et al., 2010). They have since examined carbonyl ylides (Klukovich et al., 2012), the effect of multiple transition states trapped in proximity (Lenhardt et al., 2011), and the acceleration of symmetry-forbidden transformations (Wang et al., 2015). Moving beyond *in-situ* monitoring, the Robb group developed the idea of trapping to produce multicolor soft lithography (Overholts et al., 2023), wherein reaction of a mechanochemically-generated activated furan with various secondary amines produces products of varying color.

## 4 Discussion and outlook

Both the fields of small molecule BM and PM are recognizing that *in-situ* monitoring will be vital for scale up beyond the research lab. A wealth of information is available through *in-situ* monitoring such as understanding of reaction pathways and how processing conditions alter reaction efficiencies, both of which will guide scale up (Michalchuk and Emmerling, 2022).

A wide range of TRIS methods have been developed to interrogate BM reactions such as XRD, XAFS, Raman, NMR, manometry and thermometry. These methods have been driven by characterizing small molecule transformations in the solid state to understand how these transformations can be controlled at different reactor scales.

When considering PM, the presence of the polymer chains reduces the viability of the *in-situ* BM techniques as the variety of local environments, along with the lack of crystal structure in polymer samples, makes it difficult to interpret the results of TRIS techniques such as XRD and NMR. Peterson and coworkers are examining how polymer properties such as glass transition temperature (Peterson et al., 2020) and polymer topology (Noh et al., 2023) affect mechanochemical scission in both BM and US, opening the possibility for shared understanding between the two techniques.

Large amounts of information can be gained on a single polymer chain level in SMFS but understanding how this scales into solution and the solid state is not trivial, as the host matrix influences mechanophore activation. In the solid state this introduces the need for calibration methods to allow for scale up. In solution US is not practically scalable, as energy transduction depends on reactor design and requires greater energy input at larger scales (Singla et al., 2022). As highlighted in Figure 2, in a similar move from the BM batch method to the continuous flow of TSE, examining how mechanophores are activated during nozzle flow (Willis-Fox et al., 2020; Baumann et al., 2022) shows potential for continuous mechanical activation in realistic conditions required for scale up. As with SMFS, these experiments can be coupled with computational modelling to understand mechanochemical activity (Rognin et al., 2024; Rognin et al., 2018; Rognin et al., 2021), and show great potential for combined characterization such as particle imaging velocimetry used to examine fluid dynamics (Shah et al., 2023).

The need to monitor mechanophore responses, coupled with understanding of polymer behavior, naturally requires multiple *in-situ* characterization techniques for PM in solution. In the solid state, the inhomogeneous nature of the polymer network will require calibration methods for *in-situ* PM monitoring.

When considering *in-situ* characterization techniques, it will be very important to consider the influence of off-target response within the bulk or the mechanophore itself caused by the action of the technique. For example, Saito and coworkers have noted photodegradation of the FLAP mechanophore caused by fluorescence imaging over multiple load cycles (Yamakado and Saito, 2022). Thus, further mechanophore design will be a balance of mechanical lability with stability towards characterization.

As with BM, the direction taken by *in-situ* PM monitoring will depend on the information required for scale up and the desired final application. For PM, this is less likely to be focused on reaction mechanism (versus BM) as this will have been elucidated by SMFS and computational modelling. It is more likely to be focused on understanding how mechanical force is transduced as polymer concentration scales from single molecule to solution and melt to the solid state.
